# The synergistic effect of turmeric aqueous extract and chitosan against multidrug-resistant bacteria

**DOI:** 10.1016/j.nmni.2021.100861

**Published:** 2021-03-16

**Authors:** S. Etemadi, M.H.S. Barhaghi, H.E. Leylabadlo, M.Y. Memar, A.B. Mohammadi, R. Ghotaslou

**Affiliations:** 1) Infectious and Tropical Diseases Research Center, Tabriz University of Medical Sciences, Tabriz, Iran; 2) Department of Bacteriology and Virology, Faculty of Medicine, Tabriz University of Medical Sciences, Tabriz, Iran; 3) Student Research Committee, Tabriz University of Medical Sciences, Tabriz, Iran; 4) Department of Traditional Medicine, Faculty of Traditional Medicine, Tabriz University of Medical Sciences, Tabriz, Iran

**Keywords:** Antimicrobial agent, biofilm, chitosan, multiple drug resistant, turmeric

## Abstract

We aimed to investigate the antibacterial and antibiofilm effects of turmeric and chitosan against the planktonic and biofilm forms of multidrug-resistant (MDR) bacteria. A group of MDR bacteria, including clinical isolates of methicillin-resistant Staphylococcus aureus, carbapenem-resistant Pseudomonas, carbapenem-resistant Enterobacteriaceae and AmpC-producing Enterobacteriaceae, were collected by phenotypic and genotypic assays. The broth microdilution method was used to investigate the MIC of turmeric aqueous extract and chitosan. To investigate the synergistic effect of the combination of these natural compounds, we used the checkerboard assay. According to the results of this study, turmeric and chitosan showed inhibitory effects on MDR bacteria, especially on the planktonic form of methicillin-resistant *S. aureus* as a Gram-positive compared to tested Gram-negative bacteria (carbapenem-resistant Pseudomonas, carbapenem-resistant Enterobacteriaceae and AmpC-producing Enterobacteriaceae). The antibiofilm effect of turmeric and chitosan was found more often in carbapenem-resistant Pseudomonas isolates. There was no significant difference between the tested Gram-negative bacteria because most of the tested strains were inhibited in 512 and 1024 μg/mL concentrations of chitosan and turmeric aqueous extract. In this study, turmeric aqueous extract and chitosan exhibited significant antibacterial and antibiofilm properties. However, the effect of these compounds should be investigated using in vivo models for use in pharmaceutical and disinfectant formulations.

## Introduction

Today, the emergence of multidrug-resistant (MDR) organisms such as methicillin-resistant Staphylococcus aureus (MRSA), carbapenem-resistant Enterobacteriaceae, carbapenem-resistant Pseudomonas (CRP) and AmpC-producing Enterobacteriaceae has caused serious health problems [[1], [2], [3], [4]]. Biofilm is a microbial-derived sessile community that is emanated by cells that are attached to a surface [5]. Turmeric belongs to the Zingiberaceae family and is a spice used as a food flavouring and preservative. Curcumin or diferuloylmethane is the major phytochemical, yellow and nonsoluble pigment in water and has a bioactive component isolated from the rhizome of Curcuma longa Linn., which has the chemical formula 1,7-bis(4-hydroxy-3-methoxyphenyl)-1,6-heptadiene-3,5-dione [6,7]. In the past, dried curcumin powder has been used in traditional medicine for the treatment of infections. Antitoxic, anti-inflammatory, anticancer, antimicrobial and antioxidant properties of turmeric have been reported [8,9].

Chitin is a polymer composed of *N*-acetyl glucosamine; it forms the cell wall of fungi and the exoskeletons of insects and of crustaceans such as crabs. Chitosan is a polysaccharide composed of the partial deacetylation of chitin and produced from d-glucosamine and *N*-acetyl glucosamine units linked by β-1,4 bonds [10]. It has been applied in various fields such as water purification, the food industry, paper, medicine, cosmetics and agriculture [11]. Chitosan is a highly bioactive component, and various biological activities such as cholesterol-reducing effects, free radical scavenging effects, antitumor activities, immune-stimulating effects and antimicrobial effects have been reported [12].

The aim of this study was to investigate the antimicrobial and antibiofilm effects of aqueous turmeric extract and chitosan against MDR bacteria.

## Materials and methods

### Bacterial strains

Multidrug resistance is defined as the resistance of a microorganism to three or more classes of antimicrobial agents. Forty MDR strains including ten MRSA strains, ten strains of CRP, ten strains of carbapenem-resistant *Enterobacteriaceae* (CRE) and ten strains of AmpC-producing Enterobacteriaceae were obtained from various clinical specimens. MDR strains were confirmed by phenotypic and genotypic tests [13] performed at the microbiology department of Tabriz University of Medical Sciences. In the present study, CRP involved ten clinical isolates of imipenem- and meropenem-resistant *Pseudomonas aeruginosa*. The AmpC-producing Enterobacteriaceae isolates were considered to be AmpC overproducers, while there was at a minimum a 2-fold dilution change between the MICs of imipenem and those of imipenem/cloxacillin [14]. Enterobacteriaceae isolates were tested with meropenem (10 μg) on Müller-Hinton agar plates. Incubation was performed at 35°C for 18 to 24 hours; isolates with zone diameters <25 mm to meropenem (10 μg), as stated in the European Committee on Antimicrobial Susceptibility Testing guidelines for the detection of resistance [15], were classified as CRE. Susceptibility of Staphylococcus aureus isolates to cefoxitin (30 μg) was determined by the modified Kirby-Bauer disc diffusion method following CLSI guidelines [16]. The strains of Staphylococcus aureus which were found to be resistant to cefoxitin were screened as MRSA. Escherichia coli ATCC 25922, P. aeruginosa ATCC 27853 and Staphylococcus aureus ATCC 25923 were used as quality control strains for antimicrobial susceptibility testing. This study was approved by the ethics committee of the Tabriz University of Medical Science (approval IR.TBZMED.REC.1396.891).

### Plant extraction

Turmeric rhizome was purchased from a local store, and the plant genus and species were approved by the pharmacognosy laboratory of Tabriz Drug Applied Research Center. It was washed with sterile water and cut into small pieces, then dried at 45°C for 5 days [17]. The rhizome pieces were completely powdered. To prepare the aqueous extract, 100 g of powder was dissolved in 1000 mL distilled sterilized water and boiled for 60 minutes [18]. This solution was filtered and placed inside an oven for 1 day at 45°C. Finally, the turmeric extract was stored at 4°C.

### Chitosan preparation

Chitosan was purchased from Sigma Aldrich (low molecular weight, ranging 50 to 190 kDa and 75–85% deacetylation). Chitosan solution was prepared by dissolving in 1% acetic acid. To prepare chitosan solutions, 2.5% (w/v) chitosan was dispersed in a 1.0% (v/v) acetic acid solution. The pH was adjusted to 5.8 with 10 M NaOH because the most suitable pH for solubilizing chitosan is 5.8, and this concentration lacks any antibacterial effect.

### MIC and minimum bactericidal concentration determination

To determine the MIC of turmeric aqueous extract and chitosan solution, the broth microdilution method was used on cation-adjusted Müller-Hinton broth (CAMHB). The MICs were determined according to the CLSI guideline for broth microdilution [19]. In this study, the range of turmeric aqueous extract and chitosan concentration was 32 to 4096 μg/mL. The minimum bactericidal concentration (MBC) was defined as the lowest concentration required to kill 99.9% of bacteria at incubation at 37°C for 24 hours [20].

### Checkerboard assay

Checkerboard titration is one of the methods used to investigate the interaction of antimicrobial agents. The MIC of each antibacterial agent was determined against each isolate, alone and in combination. The antibacterial effect of the combination of turmeric aqueous extract and chitosan was carried out by using 96-well microtitre plates. The formulas used to calculate fractional inhibitory concentration (FIC) indexes in the checkerboard are as follows:

1. FICA = MICA combination/MICA alone (where FICA is the FIC of drug A, MICA is the MIC of drug A in combination with drug B and MICA alone is the MIC of drug A when provided alone)
2. FICB = MICB combination/MICB alone (where FICB is the FIC of drug B, MICB is the MIC of drug B in combination with drug A and MICB alone is the MIC of drug B when provided alone)
3. FICindex = FICA + FICB (where FICindex is the sum of FICs of tested drugs)

Synergism was defined as an FIC index of ≤0.5; additive effect was defined as an FIC index of >0.5 and ≤ 1; indifference effect was defined as an FIC index of >1 and ≤ 2; and antagonism effect was defined as an FIC index of >4 [11,21].

### Quantitative detection of biofilm formation

To determine biofilm formation, the microtitre plate method was used [21]. At first, two colonies were inoculated into 5 mL Tris-buffered saline (TSB). The suspension was incubated at 37°C for 18 hours and vortexed well. It was diluted 1:100 in TSB with 1% glucose. Two hundred microlitres of solution was added to 96-well microtitre plates and incubated at 37°C for 18 hours. The culture medium with suspended bacteria was removed. The planktonic cells were aspirated, and the microplate was washed carefully three times. The plate was set upside-down and allowed to dry for 30 minutes. To stain, 200 μL of 1% crystal violet solution was added to each well for 15 minutes. After removing the colourant solution, the microplate was washed with water. The wells were permitted to dry at room temperature for 15 minutes. Then to each well 200 μL of 33% acetic acid was added. To dissolve the attached dye, it was placed at room temperature for 15 minutes. The optical density (OD) of the adherent biofilms was determined twice by the microtitre plate assay at OD 570 nm. We used TSB with 1% glucose as a negative control and biofilm-forming bacteria as a positive control.

### Determination of (fractional) biofilm inhibitory concentration

A bacterial suspension of 0.5 McFarland was prepared in TSB, and 100 μL of a suspension was added to each well of the sterile flat-bottomed 96-well microtitre plate. Then a polystyrene peg lid was placed on the microtitre plate and incubated for 20 hours at 37°C. The peg lid was washed three times, then put onto a flat-bottomed microtitre plate containing a serially diluted concentration of turmeric aqueous extract, chitosan or a combination of these in CAMHB per well, then incubated at 37°C for 20 hours. Subsequently, the peg lid was rinsed with sterile water and placed on a flat-bottomed microplate made up of CAMHB without an antibacterial compound. To transfer the biofilm from the pegs to wells, the plate was centrifuged at 805 g for 20 minutes. The peg lid was removed and the usual cover was put onto the microtitre plate. The OD was measured with the automatic microplates reader at 650 nm before and after incubation for 6 hours at 37°C. The lowest concentration of antibacterial agent whose OD650 was less than or equal to 10% of the mean of OD650 of two positive control wells present in this experiment was defined as the biofilm inhibitory concentration (BIC) [21].

## Results

### MIC and MBC determination

The MIC ranges obtained for chitosan associated with MRSA, AmpC-producing Enterobacteriaceae, CRE and CRP strains were 128–512, 256–1024, 512–1024 and 512–1024 μg/mL respectively. In addition, the MIC ranges obtained for turmeric aqueous extract associated with MRSA, AmpC-producing Enterobacteriaceae, CRE and CRP strains was 256–512, 512–1024, 512–1024 and 1024 μg/mL respectively. Of the ten MRSA strains tested, two strains of concentration 128 μg/mL, six strains of 256 μg/mL and two strains of 512 μg/mL of chitosan were inhibited. Also, out of ten strains of AmpC-producing Enterobacteriaceae, one strain of 256 μg/mL, three strains of 512 μg/mL and six strains of 1024 μg/mL of chitosan were inhibited. Regarding the ten CRE strains tested, five strains of 512 μg/mL and five strains of 1024 μg/mL of chitosan were inhibited. Of ten strains of CRP tested, four strains of 512 μg/mL and six strains of 1024 μg/mL of chitosan were inhibited. Concerning the inhibitory effect of turmeric aqueous extract, out of ten MRSA strains tested, two strains of 256 μg/mL and eight strains of 512 μg/mL of turmeric aqueous extract were inhibited. Among ten strains of AmpC-producing Enterobacteriaceae, three strains of 512 μg/mL and seven strains of 1024 μg/mL of turmeric aqueous extract were inhibited. Out of ten strains of CRE, two strains of 512 μg/mL and three strains of 1024 μg/mL of turmeric extract were inhibited. All ten CRP strains tested were inhibited by a concentration of 1024 μg/mL of turmeric extract. The average MICs obtained for chitosan in MRSA strains was 282.5 μg/mL, in AmpC-producing Enterobacteriaceae was 793.6 μg/mL, in CRE strains was 768.5 μg/mL and in CRP strains was 819.2 μg/mL. In addition, the average MICs obtained for turmeric aqueous extract for MRSA strains, AmpC-producing Enterobacteriaceae, CRE and CRP were 460.8, 870.4, 921.6 and 1024 μg/mL respectively. The MICs and MBCs obtained for chitosan and turmeric aqueous extract for the pathogens tested are presented in Table 1.

**Table 1:** MIC and MBC of chitosan and turmeric aqueous extract against MDR isolates

Antibacterial agent	Isolate	No. of strains	MIC (Range 128-1024 μg/mL)				MBC (Range 128-1024 μg/mL)			
			128	256	512	1024	256	512	1024	2048
Chitosan	MRSA	10	2	6	2	—	2	5	3	—
	AmpC	10	—	1	3	6	—	1	3	6
	CRE	10	—	—	5	5	—	—	4	6
	CRP	10	—	—	4	6	—	—	4	6
Turmeric aqueous extract	MRSA	10	—	2	8	—	—	2	8	—
	AmpC	10	—	—	3	7	—	—	2	8
	CRE	10	—	—	2	8	—	—	2	8
	CRP	10	—	—	—	10	—	—	—	10

Abbreviations: AmpC-producing *Enterobacteriaceae*; CRE, carbapenem-resistant *Enterobacteriaceae*; CRP, carbapenem-resistant *Pseudomonas*; MBC, minimum bactericidal concentration; MDR, multidrug resistant; MRSA, methicillin-resistant *Staphylococcus aureus*.

Both chitosan and turmeric aqueous extract had an inhibitory effect on MRSA compared to other tested bacteria (which had been able to inhibit bacterial growth in low concentrations). The results showed that chitosan and turmeric aqueous extract had a more inhibitory effect on MRSA strains compared to other tested bacteria because they inhibited these strains in lower concentrations. However, according to the results, there was no significant difference between the tested Gram-negative bacteria because most of the tested strains were inhibited at 512 μg/mL and 1024 μg/mL concentrations of chitosan and turmeric aqueous extract.

### Checkerboard assay

**Fig. 1:**
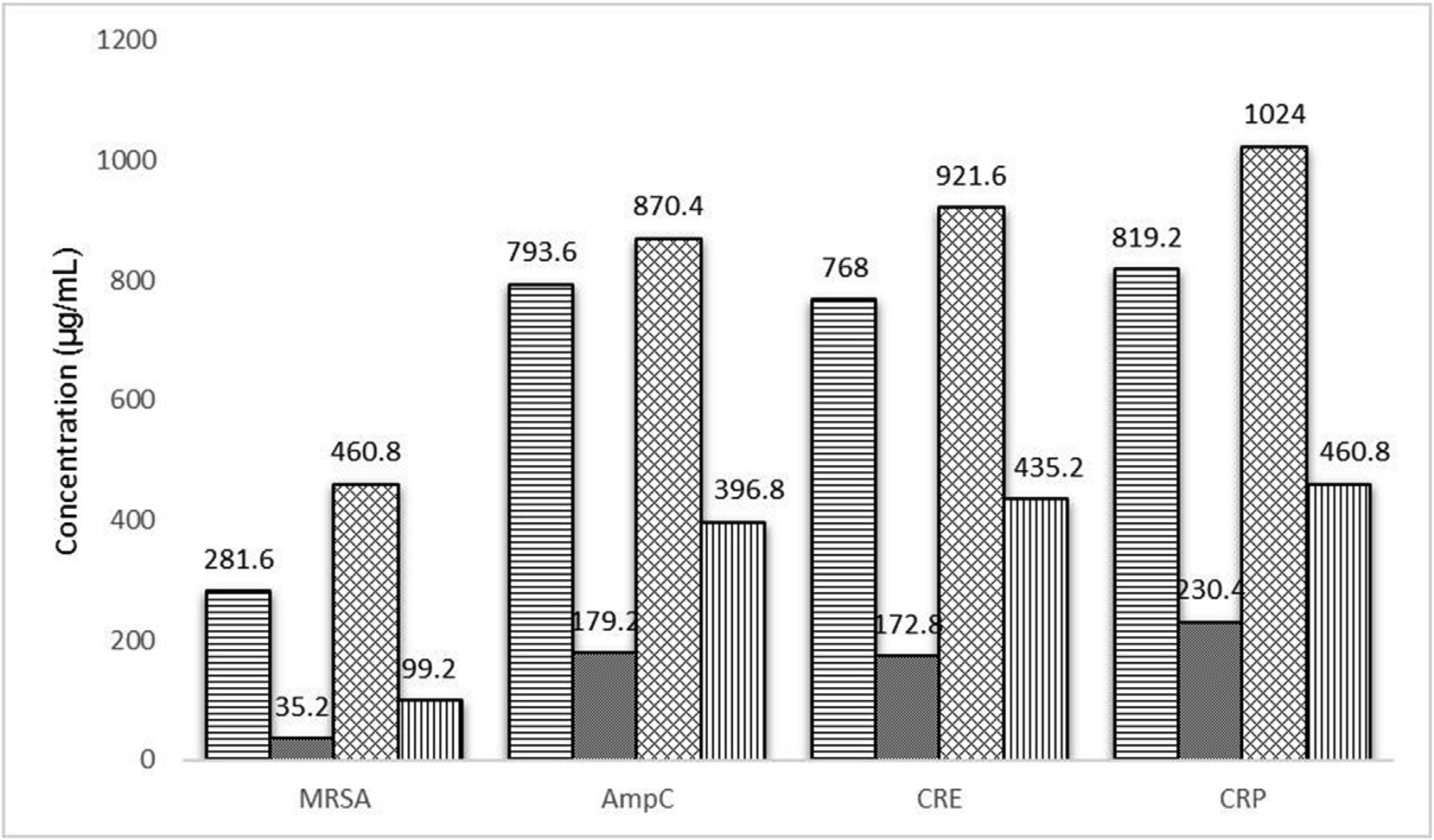
Average MIC of chitosan when used alone (), chitosan in combination with turmeric aqueous extract (), turmeric when used alone () and turmeric aqueous extract in combination with chitosan () against methicillin-resistant Staphylococcus aureus (MRSA), AmpC-producing Enterobacteriaceae (AmpC), carbapenem-resistant Enterobacteriaceae (CRE) and carbapenem-resistant Pseudomonas (CRP) isolates.

The results of the checkerboard assay showed that the combination of turmeric aqueous extract and chitosan had a synergistic effect on all ten strains of MRSA; this effect was also observed in three strains of AmpC-producing Enterobacteriaceae and two CRE strains. However, a synergistic effect was not found in the ten CRP strains tested; only an additive effect was observed. The average FIC index values obtained for the MRSA, AmpC-producing Enterobacteriaceae, CRE and CRP isolates were 0.334, 0.649, 0.674 and 0.75 respectively (Table 2). The antibacterial activity was increased in combination tests, and MICs for the combination of chitosan and turmeric aqueous extract were much lower than when used alone (Fig. 1).

**Table 2:** MICs of chitosan, turmeric aqueous extract and a combination of both compounds, and FIC indexes against MDR isolates

Isolate	MIC (μg/mL) for:		MIC in combination of chitosan and turmeric aqueous extract (μg/mL) for:		FIC
	Chitosan	Turmeric aqueous extract	Chitosan	Turmeric aqueous extract	
MRSA1	512	512	64	128	0.37 (Syn)
MRSA2	256	512	32	128	0.37 (Syn)
MRSA3	256	512	32	128	0.37 (Syn)
MRSA4	256	512	32	64	0.25 (Syn)
MRSA5	256	512	32	128	0.37 (Syn)
MRSA6	512	512	64	128	0.37 (Syn)
MRSA7	128	256	16	32	0.25 (Syn)
MRSA8	256	512	32	64	0.25 (Syn)
MRSA9	128	256	16	64	0.37 (Syn)
MRSA10	256	512	32	128	0.37 (Syn)
AmpC1	256	512	64	128	0.50 (Syn)
AmpC2	1024	1024	256	512	0.75 (Add)
AmpC3	1024	1024	256	512	0.75 (Add)
AmpC4	1024	1024	256	512	0.75 (Add)
AmpC5	512	512	128	128	0.50 (Syn)
AmpC6	1024	1024	128	512	0.62 (Add)
AmpC7	1024	1024	256	512	0.75 (Add)
AmpC8	512	1024	128	512	0.62 (Add)
AmpC9	512	512	128	128	0.50 (Syn)
AmpC10	1024	1024	256	512	0.75 (Add)
CRE1	512	1024	128	512	0.75 (Add)
CRE2	512	1024	64	512	0.62 (Add)
CRE3	1024	1024	256	512	0.75 (Add)
CRE4	512	512	128	128	0.50 (Syn)
CRE5	512	1024	128	512	0.75 (Add)
CRE6	1024	1024	128	512	0.62 (Add)
CRE7	1024	1024	256	512	0.75 (Add)
CRE8	1024	1024	256	512	0.75 (Add)
CRE9	512	512	128	128	0.50 (Syn)
CRE10	1024	1024	256	512	0.75 (Add)
CRP1	512	1024	128	512	0.75 (Add)
CRP2	1024	1024	256	512	0.75 (Add)
CRP3	1024	1024	256	512	0.75 (Add)
CRP4	512	1024	128	512	0.75 (Add)
CRP5	512	1024	256	256	0.75 (Add)
CRP6	1024	1024	256	512	0.75 (Add)
CRP7	512	1024	256	256	0.75 (Add)
CRP8	1024	1024	256	512	0.75 (Add)
CRP9	1024	1024	256	512	0.75 (Add)
CRP10	1024	1024	256	512	0.75 (Add)

FIC_index_ = FIC _chitosan_ + FIC _turmeric aqueous extract._ Synergistic effect = FIC index ≤0.5; additive effect = 0.5 < FIC index ≤1; indifference effect = 1 < FIC index ≤2; antagonistic effect = FIC index >4.

Abbreviations: Add, additive effect; AmpC, AmpC-producing Enterobacteriaceae; CRE, carbapenem-resistant Enterobacteriaceae; CRP, carbapenem-resistant Pseudomonas; FIC, fractional inhibitory concentration; MDR, multidrug resistant; MRSA, methicillin-resistant Staphylococcus aureus; Syn, synergistic effect.

### Quantitative detection of biofilm formation and BIC determination

In the present study, ten isolates were biofilm-producing bacteria, including four strains of MRSA, four strains of CRP, one strain of AmpC-producing Enterobacteriaceae and one strain of CRE. The BIC and fractional BIC (FBIC) data obtained are shown in Table 3. Both chitosan and turmeric aqueous extract at sub-MICs were able to inhibit the biofilm of the CRP strains. However, chitosan inhibited the biofilm formation in AmpC-producing Enterobacteriaceae and CRE in a concentration below the MIC but on the biofilm produced by MRSA strains at a concentration above the MIC levels found to be effective. Turmeric aqueous extract also affected MRSA, AmpC-producing Enterobacteriaceae and CRE isolates at concentrations higher than MIC. Regarding the combined effect of the two substances of interest, it should be noted that only the biofilm produced by CRP exhibited a synergistic effect.

**Table 3:** Biofilm inhibitory concentrations of chitosan, turmeric aqueous extract and the combination of both compounds and FBIC indexes against MDR isolates

solate	BIC (μg/mL) for:		BIC in combination of chitosan and turmeric aqueous extract (μg/mL) for:		FBIC
	Chitosan	Turmeric aqueous extract	Chitosan	Turmeric aqueous extract	
MRSA1	4096	2048	2048	512	0.75 (Add)
MRSA3	4096	2048	2048	512	0.75 (Add)
MRSA4	4096	2048	2048	256	0.62 (Add)
MRSA5	4096	2048	2048	512	0.75 (Add)
CRP1	128	512	32	128	0.50 (Syn)
CRP4	256	512	64	64	0.37 (Syn)
CRP6	256	512	64	128	0.50 (Syn)
CRP7	256	512	64	128	0.50 (Syn)
AmpC6	512	4096	64	2048	0.62 (Add)
CRE8	512	4096	64	2048	0.62 (Add)

Abbreviations: Add, additive effect; AmpC-producing *Enterobacteriaceae*; BIC, biofilm inhibitory concentration; CRE, carbapenem-resistant *Enterobacteriaceae*; CRP, carbapenem-resistant *Pseudomonas*; FBIC, fractional biofilm inhibitory concentration; MDR, multidrug resistant; MRSA, methicillin-resistant *Staphylococcus aureus*; Syn, synergistic effect.

## Discussion

In the current study, the results of the MICs and MBCs of chitosan on MDR bacteria indicated that chitosan had an inhibitory effect on all tested bacteria, although the inhibitory effect of chitosan on MRSA is higher than on Gram-negative bacteria. A study examined whether the ethyl acetate extract of C. longa could diminish the MICs of β-lactams by the checkerboard dilution method and showed that the C. longa extract markedly lowered the MICs of ampicillin and oxacillin against MRSA. All strains saw a 2- to 16-fold reduction in the MICs [18]. Mun et al. [6] reported that curcumin, a compound in turmeric, had strong antimicrobial activities and synergistic effects when used alone (MIC 125–250 g/mL) as well as when used in combination with antibiotics (OXI, AMP, CIP, NOR) in all the S. aureus strains tested. Our results are consistent with some previous studies that have suggested that chitosan has a better inhibitory effect on Gram-positive bacteria than on Gram-negative bacteria [22,23]. Various studies have demonstrated that curcumin is active against Gram-negative bacteria like E. coli and the formation of its biofilms, while both effects are enhanced by curcumin nanoparticles [24,25]. In addition, the antibacterial effects of curcumin were detected against E. coli and Salmonella enterica serotype Typhimurium in the 1980s [26].

Regarding the effect of chitosan on the biofilm of the tested bacteria, chitosan showed a better effect on the Pseudomonas spp. and Enterobacteriaceae biofilms, which was the opposite of what was found in the planktonic cells. The results of this study showed that chitosan had the lowest inhibitory effect on S. aureus biofilm; the highest resistance to chitosan was attributed to the MRSA biofilm. The BICs of chitosan for MRSA were 8- to 16-fold that of planktonic cells. In addition, the combination of turmeric aqueous extract and chitosan did not significantly reduce the BICs of chitosan on MRSA biofilm compared to chitosan individually. While BICs of chitosan decreased as a result of the addition of turmeric aqueous extract in AmpC-producing Enterobacteriaceae, CRE and CRP isolates to one-fourth to one-eighth the BICs of chitosan alone. Overall, the response of S. aureus biofilm to antibiotics is lower than planktonic cells (4- to 512-fold less than that of planktonic cells) [27]. In one study, chitosan was shown to inhibit the biofilm of MRSA in both biofilm formation and mature biofilm; however, the inhibitory effect on mature biofilm has been reported less often than biofilm formation [10]. In another study, the effects of chitosan in three concentrations of 0.01%, 0.1% and 1% on mature bacterial biofilms were investigated. The study indicated that with a concentration of 1% chitosan (the highest concentration), the biofilm of Listeria monocytogenes was inhibited more than others, followed by Pseudomonas spp., Salmonella spp., Bacillus spp. and S. aureus. Various factors may affect the effect of chitosan on biofilms of different bacteria. Chitosan is a cationic biopolymer, which is positively charged by the presence of amine groups, whereas biofilm exopolysaccharide in some bacteria such as Pseudomonas spp. is polyanionic, and in Staphylococcus spp., adhesions are polycationic. The biofilm matrix structure can also be another explanation; the biofilms of Pseudomonas spp. are thin and highly susceptible. However, chitosan properties such as molecular weight, degree of deacetylating and concentration on the penetration into biofilm matrix can be affected [28].

Our results indicate that turmeric aqueous extract had an antibacterial effect on MDR-tested bacteria. The antibacterial activity of turmeric is due to the presence of various compounds such as valeric acid, turmerol, essential oil, curcumin and an alkaloid in its structure. Many studies have been conducted to investigate the antimicrobial effect of turmeric aqueous extract [18,29]. The results of our study are in line with their results. The important point to be noted is the greater inhibitory effect of turmeric on MRSA as Gram-positive bacteria rather than Gram-negative bacteria. The reason for this could be the difference in the structure of the bacteria and the cell wall in Gram-positive and Gram-negative bacteria [29]. Our study, like some previous studies, showed that curcumin had an inhibitory effect on the biofilm of P. aeruginosa, S. aureus and Enterobacteriaceae [30,31]. Considering the antibacterial and antibiofilm effect of curcumin on P. aeruginosa, we suggest that the effect of curcumin on P. aeruginosa may vary depending on the strain. One study reported that curcumin inhibited biofilm formation in clinical isolates of E. coli, S. aureus and P. aeruginosa [31]. The differences in results may be due to the characteristics of the bacterial strain. In the current study, MDR bacteria were used, and MDR bacteria are more resistant to antibiotics. The turmeric aqueous extract was used in a water solvent instead of a dimethyl sulphoxide solvent, and our study was based on mature biofilm, not on biofilm formation. The combination of curcumin and chitosan together with aloe vera inhibited the growth of microbes in wool, cotton and rabbit hair, and they can be used as an antimicrobial agent in the textile industry [32].

The exact antibacterial mechanism of chitosan remains ill described, but various mechanisms have been proposed. Chitosan has receptive, positively charged amino groups that can associate with the negatively charged bacterial cell membranes, resulting in the leakage of proteinaceous and other intracellular constituents and a modification of cell permeability [33]. The promising results of curcumin's antimicrobial activity made it a good candidate to enhance the inhibitory effect of existing antimicrobial agents through synergism [34]. It was found that curcumin decreases the bundling of FtsZ protofilaments related to the limiting binding ability to FtsZ with a separation constant of 7.3 μM. It showed that curcumin, through inhibition assembly dynamics of FtsZ in the Z ring, may possibly suppress bacterial cell proliferation as a plausible antibacterial mechanisms of action. Examination of E. coli and Bacillus subtilis showed that curcumin, by its inhibitory impact against FtsZ polymerization, could suppress FtsZ assembly, leading to disruption of prokaryotic cell division [35]. In the present study, the combination of chitosan and turmeric aqueous extract had a synergic effect on the planktonic and biofilm forms of the bacteria, and a greater effect was observed on planktonic bacteria of MRSA and biofilm-forming CRP.

Despite these promising results, our study had limitations such as the lack of scanning electron microscopy and confocal laser scanning microscopy to further explore the biofilms. To better understand the function of chitosan, turmeric and their combination in inhibiting MDR bacteria, molecular and genomic research is required. Further investigation is required to evaluate the antibacterial activity of turmeric aqueous extract for the eradication of bacteria and the improvement of health; also, the antimicrobial mechanisms of turmeric aqueous extract require more study. In future studies, it is recommended to test the effects of these compounds on animal models.

## Conclusion

The results of this study demonstrate that turmeric and chitosan substances have an in vitro inhibitory effect on the planktonic and biofilm forms of MDR bacteria. Further examination is needed to completely understand turmeric aqueous extract and chitosan to improve formulations that will make it usable as a drug.

## Conflict of interest

None declared.
